# Searching smarter, not harder: leveraging AI to enhance literature searches for theory-driven reviews—A methodological case study

**DOI:** 10.1186/s12874-026-02814-3

**Published:** 2026-03-04

**Authors:** R. Hunter, A. Booth, L. Wood

**Affiliations:** 1 Department of Public Health and Sports Sciences, Medical School Building, University of Exeter, St Luke’s Campus, Exeter, EX1 2LU UK; 2 School of Communities and Education, Northumbria University, Coach Lane Campus, Newcastle Upon Tyne, NE7 7XA UK; 3 School of Health and Related Research (ScHARR), The University of Sheffield, Sheffield City Centre, 30 Regent St, Sheffield, S1 4DA UK

**Keywords:** Literature searches, Review methodology, Realist reviews, Evidence appraisal, Literature screening, Artificial intelligence

## Abstract

**Background:**

Integrating artificial intelligence (AI) into literature searching has the potential to enhance research synthesis by improving the identification of conceptually rich or otherwise difficult-to-locate evidence. Theoretical or conceptual literature reviews, including realist reviews, often involve resource-intensive searches because they aim to trace nuanced ideas, mechanisms, or conceptual relationships across multiple sources. This case study illustrates the use of AI-powered tools to support and streamline such literature searching, using a realist review as an example.

**Methods:**

We applied AI tools—Scite and Undermind—in the context of a realist review to facilitate the identification of relevant studies. Seed papers and key informant papers guided the search, and a novel classification system (grandparent, parent, and child papers) was used to systematically organise studies for developing and refining theoretical constructs. Transparent screening procedures and decision-making frameworks were employed to ensure methodological rigour and reproducibility.

**Results:**

The integration of AI tools supported the retrieval of conceptually relevant literature and helped manage complex datasets. The classification system enabled structured organisation of studies, supporting iterative testing and refinement of theoretical constructs. The workflow demonstrated flexibility and adaptability, suggesting potential applicability beyond realist review.

**Conclusions:**

Our findings suggest that AI-powered tools can support literature searching, particularly in identifying conceptually relevant studies. However, these tools do not replace the critical interpretive work required by researchers. Human judgement remains essential to assess relevance, evaluate nuanced concepts, and make informed decisions throughout the search process, with AI serving as a valuable adjunct rather than a substitute.

**Supplementary Information:**

The online version contains supplementary material available at 10.1186/s12874-026-02814-3.

## Background

The advancement of artificial intelligence (AI) capabilities presents an opportunity to enhance research processes, particularly in literature searching [1]. Capturing nuanced concepts, identifying theoretically relevant papers, or tracking related studies can be time- and resource-intensive using conventional methods. AI-powered tools offer opportunities to streamline these processes while maintaining conceptual depth and rigour. This is particularly relevant for literature reviews with a theoretical or explanatory focus, such as realist reviews.

Realist reviews aim to understand complex interventions by examining how context interacts with underlying mechanisms to produce outcomes [2]. To do this, they often require broad, inclusive searches to identify conceptually rich material to develop and test programme theories [3]. The “realist search” must be systematic, transparent, and guided by established methodological principles yet also iterative, fluid, and purposeful [4–7].

Instead of relying on pre-specified topic-based procedures, the realist search embraces multiple diverse retrieval techniques throughout the review [4]. Berry picking [8], by which the searcher ‘forages’ using six search techniques: checking cited references; citation searching; searching relevant journals; browsing proximate to items of interest; using subject-indexed databases; and author searching [8] has enjoyed a renaissance in the specific realist guise of CLUSTER Searching. Specifically, CLUSTER searching constitutes a systematic methodology for identifying a study cluster (a group of related reports from a single project) by tracking Citations, Lead Authors, Unpublished materials, Scholar searches, Theories, Early examples, and Related projects [5]. More generally, Snowballing or Citation Chaining offers a method for following up citations of a key study either backward (reviewing references) or forward (tracking subsequent citing articles) to find contextually or theoretically proximate documents [7].

Beyond citation-based methods, realist searches often incorporate alternative sources and targeted searches. Grey Literature searching seeks out non-peer-reviewed sources, such as policy documents, reports, web-based evaluations, and manuscripts, to supply the necessary theoretical or contextual detail missing from conventional literature [9]. More broadly, the use of general search engines (e.g., Google) to retrieve grey literature and discover programme theories has been suggested to be more efficient than academic database searches for theory identification [9] while scholarly Search Engines such as Google Scholar offer comprehensive coverage, citation analysis, or identification of full-text documents [5]. Meanwhile, focused named Theory Searches (e.g., using a Behaviour, Health context, Exclusions, Models or Theories—BeHEMoTh—template) specifically target literature that explicitly mentions or discusses relevant mid-range theories [10].

While these techniques have proven valuable, they present practical challenges. Manual implementation of multiple search strategies is labour-intensive and time-consuming [11]. Citation chaining requires extensive manual tracking across multiple sources and document types [5]. Identifying semantically related concepts—particularly the nuanced theoretical constructs central to realist reviews—remains difficult using keyword-based searching alone [12]. Assessing relevance across large volumes of retrieved literature demands significant expertise and repeated judgement calls [13]. These limitations can constrain the breadth and depth of evidence synthesis, particularly for complex interventions where relevant insights may be scattered across diverse literatures.

Recent advances in AI and large language model (LLM)-assisted tools offer potential solutions to these challenges. These tools can identify relevant studies through semantic understanding rather than keyword matching, support automated relevance ranking based on theoretical alignment, and streamline citation tracking and cross-referencing processes [14, 15]. AI applications are now capable of automating multiple stages: generating tailored search strategies, conducting iterative searches that adapt based on emerging findings, and directly identifying studies based on semantic similarity [14, 15].

However, the rapid development of innovative literature search tools—particularly those powered by AI—has created an emerging need for contemporary methodological guidance to help researchers effectively utilise these tools within established review frameworks. While AI capabilities offer clear potential to address the practical challenges of realist searching, there is limited guidance on how to integrate these tools while maintaining the systematic, transparent, and theoretically grounded approach that realist methodology requires. This case study aims to address this need by providing a detailed, replicable account of how AI-driven tools can be integrated into the realist review process, ensuring that research methodologies evolve alongside technological advancements.

Although developed in the context of a realist review, the underlying process—systematically extending searches to surface evidence that conventional strategies may overlook—has broader applicability. Any literature review seeking to identify material not easily captured through standard indexing or keyword-based searching could benefit from this approach. By clearly outlining the steps taken to retrieve and select relevant studies, we seek to enhance transparency and replicability, making it easier for others to follow. In doing so, this case study provides a detailed, replicable account of how these advanced tools were integrated into the realist review process. The goal is not only to demonstrate how these innovative methods can be used effectively but also to encourage other researchers to adopt and apply these approaches in their own reviews, contributing to the ongoing evolution of the field.

## Methods

Realist review is a flexible approach to evaluating the literature, allowing for variation in how the process is undertaken [16]. While authors may perform more or fewer steps than those outlined by Pawson, his framework consists of six key stages: identifying the review question, searching for empirical evidence, quality appraisal, data extraction, data synthesis, and dissemination of findings [2]. This study focuses specifically on steps two and three—searching for empirical evidence and conducting quality appraisal—within the context of a realist review evaluating prehabilitation programmes for individuals awaiting spinal surgery for neurogenic claudication (NC) [17]. We describe the use of AI-powered tools, Scite and Undermind (among others available), to identify primary studies for testing Context-Mechanism-Outcome Configurations (CMOCs) developed as part of a programme theory. The following sections outline the search process and provide a transparent and rigorous approach to screening results. We present this process, not as a prescriptive method or a model of "best practice," but rather as a resource for other researchers to adapt to their own studies.

### Searching for empirical evidence

Our search focused on identifying empirical studies to test, refine, and potentially refute the CMOCs developed within an initial programme theory —a preliminary framework outlining the expected relationships between context, mechanisms, and outcomes for prehabilitation in NC surgical candidates. Further details on the development of the initial programme theory and the realist review methods have been published elsewhere [18]. This objective shaped our search strategy and influenced the selection of tools used. Guided by an experienced information specialist with expertise in realist methodology (AB), the research team decided to use two AI-powered tools (Scite and Undermind) with complementary functions. Scite was used to explore citation networks and analyse how concepts were cited across the literature—identifying supporting, contrasting, or mentioning evidence through its claim-analysis capabilities. Undermind was used to conduct semantic searches that identified conceptually relevant papers through natural language processing, even when specific terminology differed (see Table 1 for further details on Scite and Undermind). Using these tools together enhanced our ability to retrieve studies that conceptually aligned with the CMOCs under investigation Although we chose to use Scite and Undermind specifically,, the methods we describe are not dependent on these particular tools; the principles outlined here are broadly applicable and can be operationalised using other similar platforms.

**Table 1:** Overview of scite and undermind—purpose and capabilities [19]

**Scite: Claim verification and citation analysis**
• Claim Verification: Assesses scientific claims by analysing how they have been supported, contradicted, or mentioned across the literature • Citation Analysis: Tracks how individual papers have been cited, offering insight into their influence and relevance • Research Tool Integration: Compatible with Google Scholar, PubMed, and Web of Science • Output: Provides contextual citation insights to enhance the interpretation of evidence
**Undermind: Advanced literature searching for complex or interdisciplinary topics**
• Iterative Search Process: Refines and expands search strategies in real time to uncover both central and peripheral literature • Semantic Understanding: Uses natural language processing to grasp the meaning behind research questions and extract conceptually relevant studies • Personalised Recommendations: Adapts search results to match a researcher's specific interests and goals • Output: Yields a broader and more nuanced evidence base tailored to the review’s evolving needs

#### Search strategy

Scite's claim-analysis capabilities, which classify citations as ‘supporting,’ ‘contrasting,’ or ‘mentioning’ the claims of the cited work [20], were used to explore the core concepts within each CMOC. Targeted search queries, combining 'prehabilitation' with relevant keywords, were constructed (see supplementary file 1 for an example). This approach enabled the research team to analyse how concepts were used and referenced within the literature—specifically, the 'citation context'—allowing the research team to identify supporting or contrasting evidence and assess the credibility and application of these concepts within the prehabilitation literature.

To maintain precision in our search, we privileged retrieval of the most relevant studies rather than expanding the search with numerous synonyms or alternative terms. For example, to test CMOC1, the search strategy used was: Prehabilitation "waiting list" OR Prehabilitation "waiting period". We chose these terms deliberately, as we considered it unlikely that a relevant full-text document would fail to reference either of these terms if it were addressing prehabilitation in waiting lists or periods. Although additional synonyms could have broadened our results, this focused strategy helped ensure that the studies retrieved were directly relevant to our research question. Such an approach contrasts with systematic reviews where a review team works painstakingly through a list of terms of increasing futility.

Undermind generated detailed reports on specific research topics aligned to each CMOC. For our search related to CMOC 1, we began by copying and pasting the CMOC into the initial query box of Undermind, which then guided us through a series of answer-and-response prompts to clarify the focus of our search. Through this iterative process, Undermind helped clarify the key concepts of interest, such as the type of surgery and the desired outcomes. After refining these details, Undermind generated a final search query for CMOC 1: ‘*The impact of prehabilitation on patient empowerment and stress reduction in individuals awaiting orthopaedic surgeries’*. Based on this, Undermind produced a comprehensive report for CMOC 1, summarising relevant literature related to key concepts such as patient empowerment, stress reduction, multimodal programmes, and orthopaedic prehabilitation. The report also included curated citations, helping to streamline the identification of contextually relevant studies. (see supplementary file 2 for an example of a report).

#### Initial screening

Having retrieved a set of potentially relevant papers from both Scite and Undermind, the next step involved systematically screening these results to assess their relevance to the CMOCs. To reduce the volume of papers and make the dataset manageable, preliminary screening of the literature results was conducted by a single researcher (RH). Although this stage was not formally quality assessed, it was guided by broad criteria for assessing relevance, richness, and rigour (RRR) [21]. The RRR framework is widely used in realist research to evaluate studies across these three dimensions rather than focusing solely on methodological rigour as traditional appraisal tools do. This recognises that valuable insights ('nuggets') contributing to theory development can be found even in studies that may be considered methodologically weak by traditional standards. The Undermind search engine assigned a percentage score to each paper, indicating how closely it matched the research query. Papers scoring below 50% were excluded from further consideration. Among those scoring above 50%, Undermind highlighted ‘top scoring’ papers it considered key. The research team reviewed these to verify alignment with the CMOC and then applied the RRR framework to determine which papers should proceed to full-text review.

This initial screening process evokes what Bates described as ‘berrypicking’—a model of real-world information seeking where queries evolve over time and researchers gather relevant material iteratively [8]. Just as a forager scans multiple bushes, choosing only the most promising berries, this stage involved scanning AI-ranked results and selectively “picking” studies based on both algorithmic cues and the RRR framework. ‘Berrypicking’ aligns well with the interpretive, non-linear nature of realist reviews, where studies are selected not through exhaustive retrieval but through theoretically informed, context-sensitive filtering [5]. The research team concluded that the risk of exclusion errors at this stage was minimal. Any borderline decisions would be captured in subsequent full-text screening phases, and the grandparent-parent–child approach (described below) meant that conceptually relevant literature could still be retrieved through citation chaining even if initially missed at this stage.

In our review, the number of papers retrieved from Scite for each CMOC was generally small (1–7 papers) due to the conceptual specificity of each CMOC. As such, all retrieved papers were taken directly to full relevance assessment. Reading Scite’s citation statements—brief extracts from the full text highlighting the relationship between a cited study and the citing paper—helped confirm the relevance of these studies to the CMOC. In scenarios where Scite returns larger sets of papers, citation statements could support a staged prioritisation approach that could be applied consistently across all CMOCs, enabling researchers to focus first on mechanism-rich studies while maintaining consistency and rigour. Even if larger numbers of papers were retrieved, it is plausible that reviewing and selecting relevant studies would be faster using the citation statements than relying solely on titles and abstracts in traditional searches. This, however, remains conjectural and warrants further investigation.

### Application example: initial screening of results for CMOC 1

**Fig. 1:**
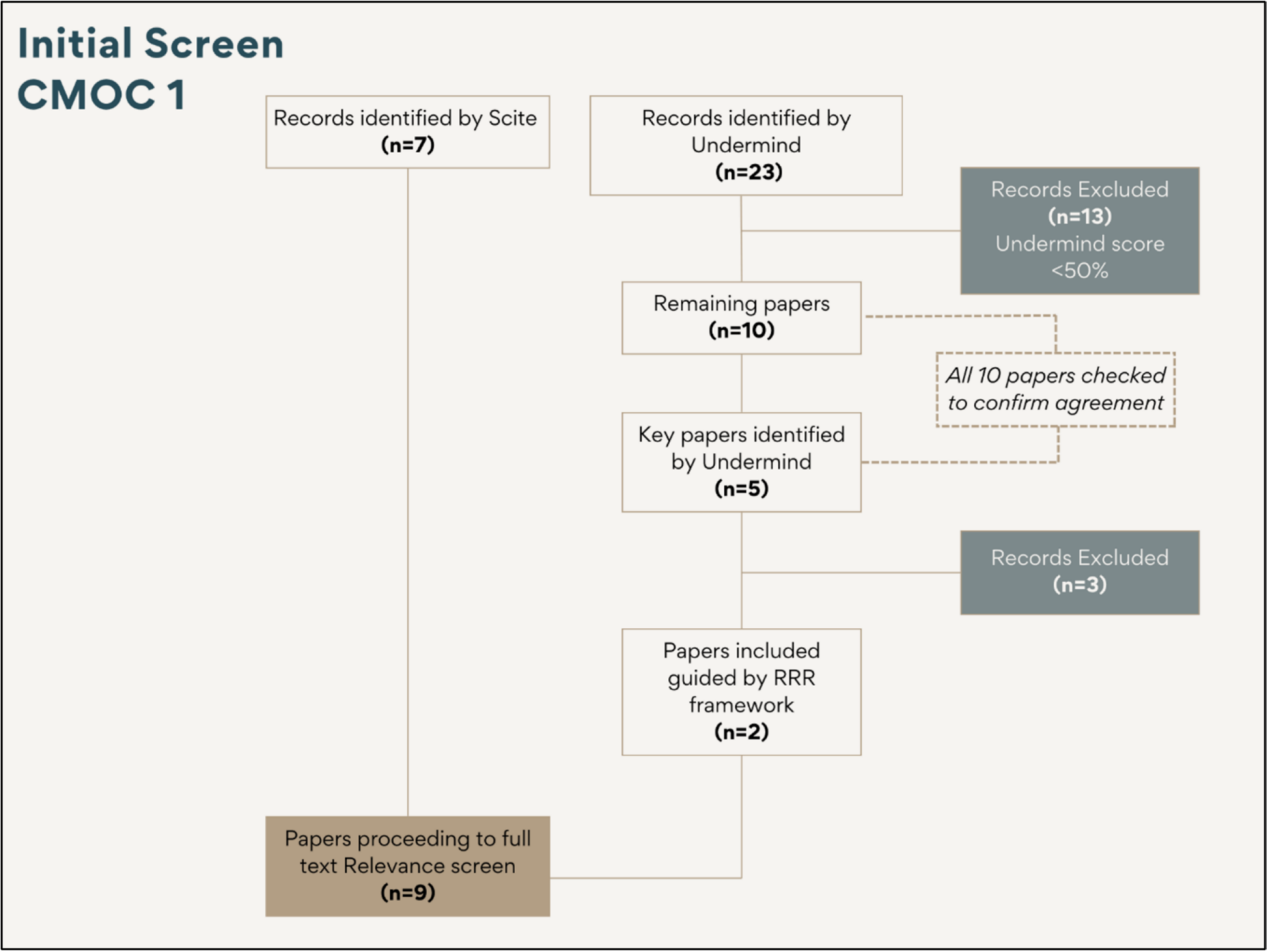
An initial screen of search results for CMOC 1

In the case of CMOC 1, Scite produced seven results —considered a manageable number—which were taken forward directly without the need for initial screening. In contrast, Undermind presented 23 results, of which 13 scored below the 50% threshold. After a brief review of titles and summaries, these 13 papers were excluded. Of the remaining ten, Undermind identified five papers as key. While these five were prioritised, the researcher reviewed all ten to validate Undermind’s assessment and ensure no relevant studies were overlooked. The RRR framework was then applied to the five key papers resulting in two being selected for full-text relevance screening. These two papers were then combined with the seven identified by Scite, resulting in a total of nine papers to be taken forward to a full text relevance screen (See Fig. 1 for illustration).

### Seed and key informant papers

To support the iterative nature of the review and maintain a transparent record of potentially relevant evidence, studies were categorised as *seed papers* or *key informant papers*. A *seed paper* refers to a secondary study that was not included in the analysis itself but cited primary studies that may be relevant to one or more CMOCs. The use of this term was informed by the Terminology, Application, and Reporting of Citation Searching TARCiS statement, which defines “seed references” as relevant articles known beforehand and used as a starting point for citation searches [22]. Adopting this label allowed for systematic tracking of secondary studies that served as gateways to further primary research. *Key informant papers*, on the other hand, were primary studies that were directly relevant to more than one CMOC within the programme theory (See Table 2 for a definition of papers). This coding enabled the research team to ensure that studies with the potential to inform multiple CMOCs were flagged for further analysis, ensuring a thorough and connected synthesis across the programme theory.

**Table 2:** Definition of paper types

Paper Type	Definition/Decision Criteria
Seed paper	A secondary study not included in the review itself but which cites primary studies that may be relevant to the programme theory’s CMOCs
Key informant paper	A primary study that is relevant to more than one CMOC
Grandparent Paper	• Primary or secondary studies identified in the initial screen as being potentially relevant to the CMOC being tested • If the grandparent paper is a primary study and meets the relevance criteria, it is included in the second formal screening phase for Relevance, Richness, and Rigour (RRR) • If the grandparent paper is a secondary study, it is not included in the review • If the grandparent paper is a secondary study that references potentially relevant primary studies informing the CMOC being tested or other CMOCs, it is colour-coded as a *seed paper* and tagged with hashtags for each CMOC it may inform • If the grandparent paper is a primary study that does not directly inform the CMOC being tested but is relevant to other CMOCs in the programme theory, it is also categorised as a *key informant* and similarly tagged
Parent paper	• A primary study identified from a grandparent paper that is considered potentially relevant for the CMOC being tested or for other CMOCs within the programme theory • If a parent study is relevant to more than one CMOC, it is coded as a key informant paper and assigned hashtags for each CMOC it is considered potentially relevant to
Child paper	• A primary study identified from an included parent paper that is considered potentially relevant for the current context-mechanism-outcome configuration (CMOC) being tested

#### Relevance screen

After the initial screen, papers identified from the search results of Scite and Undermind as potentially relevant were categorised as ‘grandparent papers’ and subjected to a full-text relevance screen. This process involved assessing whether the studies directly addressed key concepts and variables within the CMOC being tested, as well as evaluating their potential to inform other CMOCs. As outlined in the criteria below (see Table 3), studies were classified based on their relevance to the CMOC being tested, with additional considerations for those that could inform multiple CMOCs.

**Table 3:** Relevance screening and categorisation criteria for CMOC analysis: Include, exclude, and inform

Include	• Primary studies (qualitative or quantitative) that are directly relevant to the CMOC being tested, meaning the study explicitly addresses concepts or variables within the CMOC • Primary studies that are relevant to the CMOC being tested but also identified as relevant for other CMOCs. Studies with dual relevance will be included in the current CMOC analysis and coded as a "key informant" for future re-evaluation with other CMOCs • Primary studies not relevant to the current CMOC, but relevant to other CMOCs: These studies, will be coded as key informants for other CMOCs and retained for future analysis
Exclude	• Primary studies that are not related to the CMOC being tested or any other CMOC within the programme theory • Secondary studies that are not relevant to the CMOC being tested or any other CMOC within the programme theory
Inform	• Seed papers: Secondary studies (e.g., reviews, meta-analyses) that cite primary studies potentially relevant to the CMOC being tested or other CMOCs within the programme theory. These papers will be reviewed to identify relevant primary studies • Key informant papers: Primary studies that are not directly relevant to the CMOC being tested but may be relevant to other CMOCs within the programme theory. These papers will be flagged for future re-evaluation. If a study becomes relevant to a CMOC already tested, it will be re-evaluated for inclusion

### Grandparent papers

**Fig. 2:**
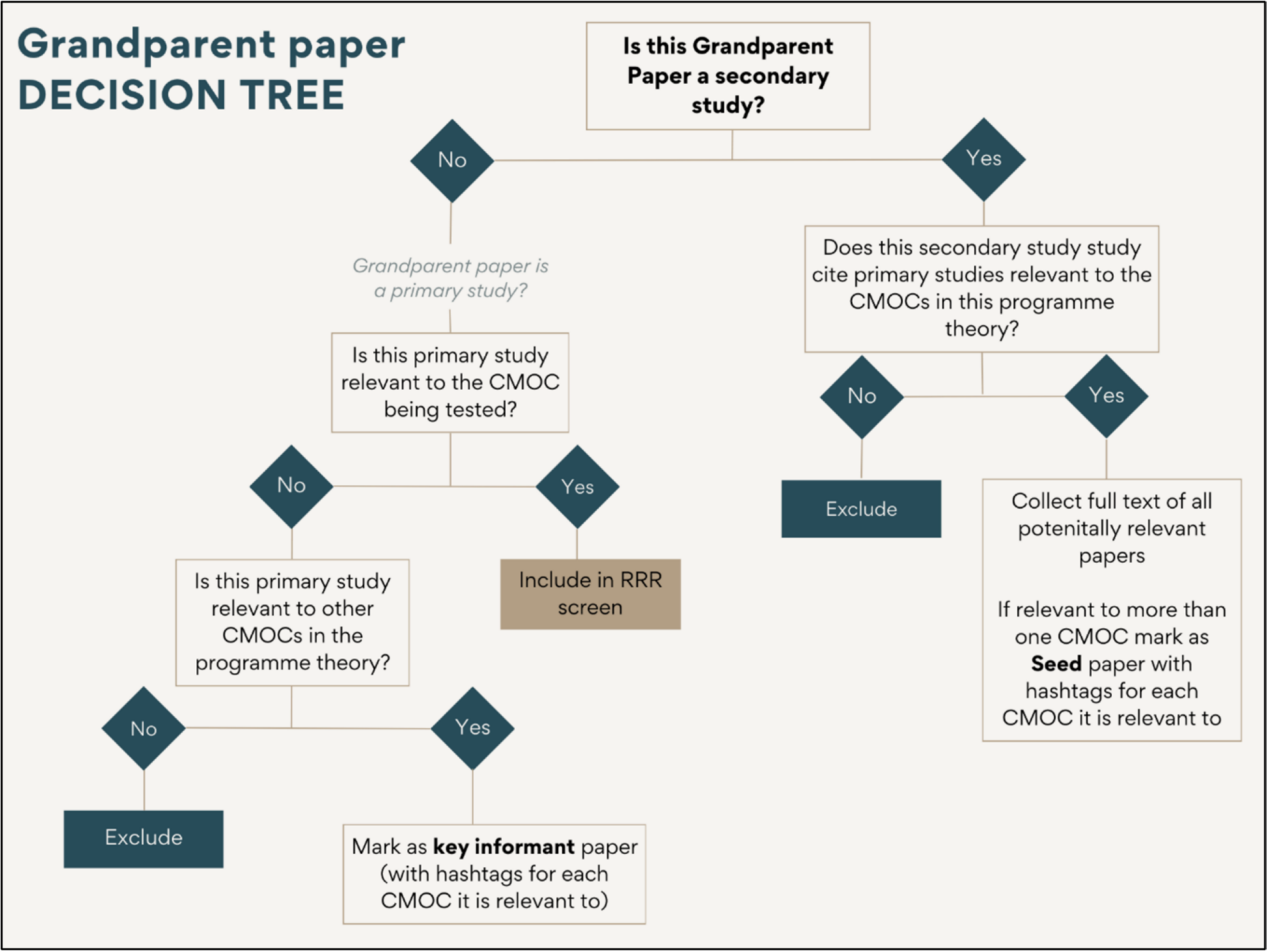
Decision tree for evaluating grandparent paper

Since our literature search was specifically designed for searching for empirical evidence for testing CMOCs [2], only primary studies were considered for inclusion at this stage. Relevant grandparent studies (studies retrieved from the AI systems) that were primary studies advanced to RRR screening. Grandparent studies classified as secondary studies, retrieved similarly via the AI systems, were not includable in the review in their own right but were mined for any potentially relevant primary studies. If relevant primary studies were identified, the grandparent study was colour coded to indicate its status as a ‘seed paper’. This process continued until all relevant grandparent studies for the CMOC under testing had been screened. Figure 2 illustrates the decision tree to guide the researcher in determining the appropriate course of action regarding grandparent papers.

### Application example: grandparent paper relevance screen CMOC 1

**Fig. 3:**
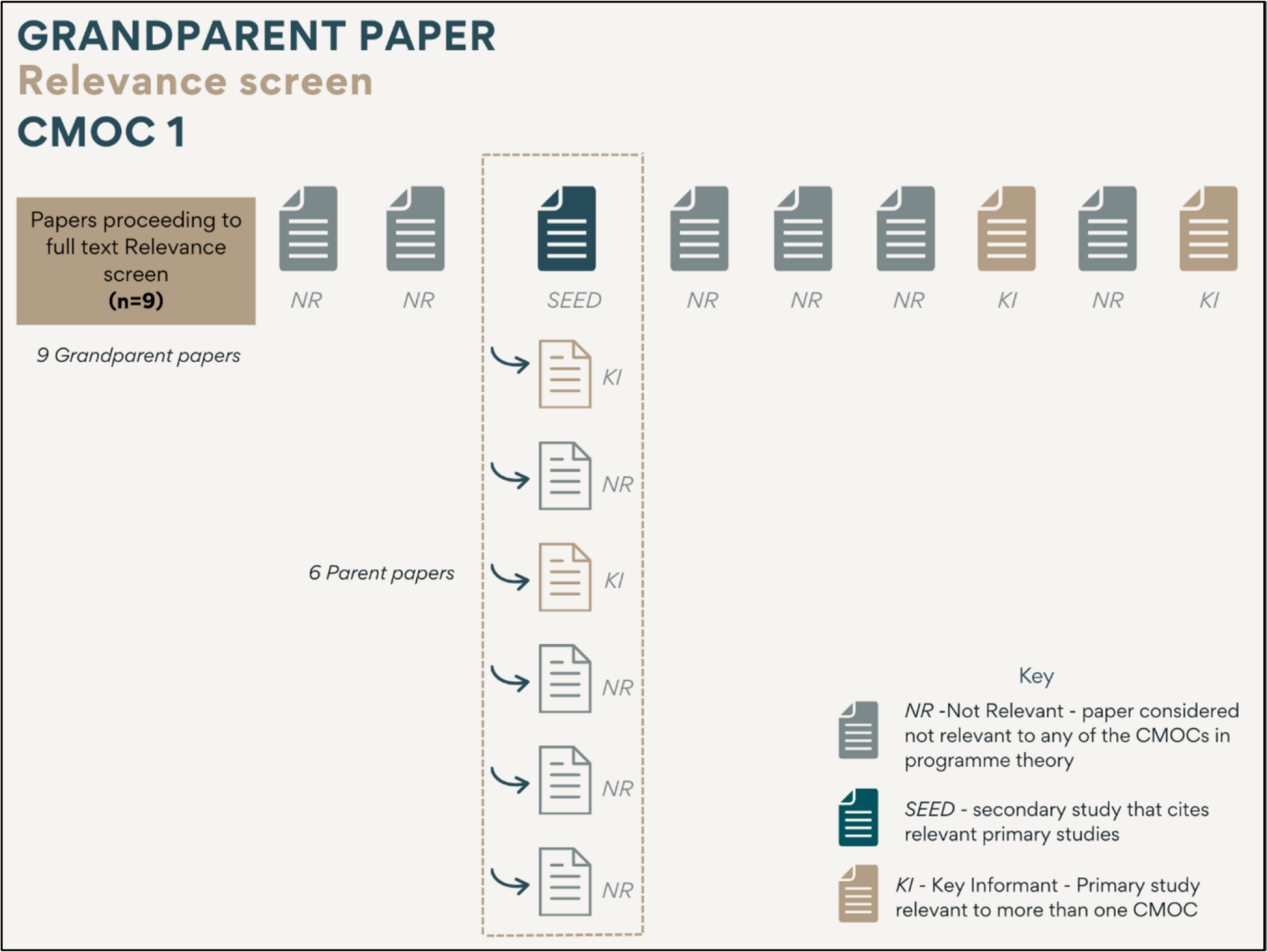
Overview of relevance screen for CMOC 1

Using CMOC 1 as an example, nine papers were identified from the initial screen as potentially relevant to CMOC 1 (see Fig. 3). These were read in full and screened for relevance both for CMOC 1 and for any other CMOCs in the programme theory. Six of the nine papers were considered not relevant and were excluded (see Table 3 for exclusion criteria). One paper was a secondary study which referenced potentially relevant primary studies, and thus it was coded as a seed paper. Two papers were primary studies that were not relevant to the current CMOC being tested but were considered relevant to other CMOCs in the programme theory, and so they were coded as key informant papers.

### Parent papers

**Fig. 4:**
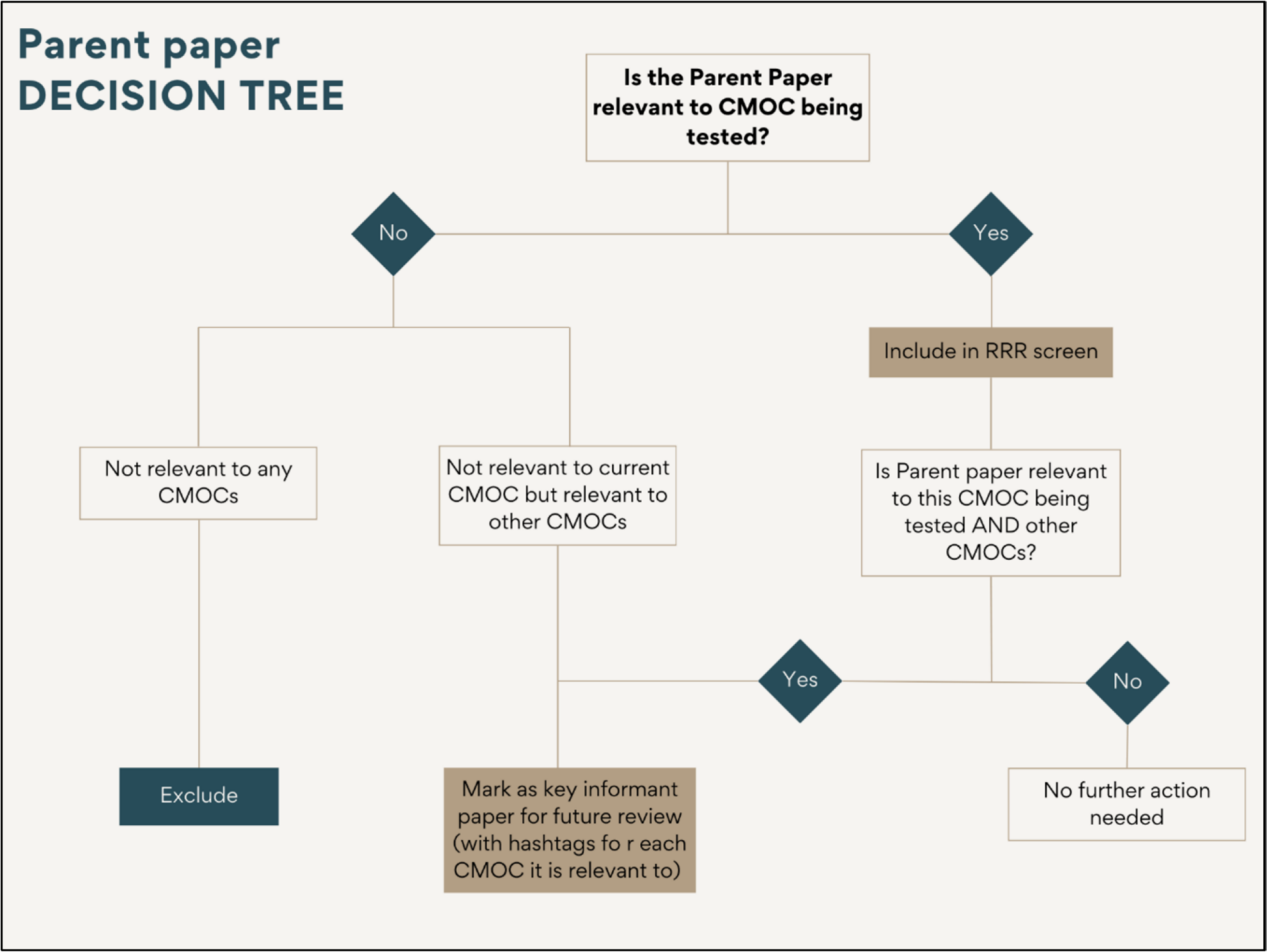
Decision tree for screening and categorising parent papers

Relevant primary studies identified from the grandparent papers were then, in turn, termed *parent papers*. Each parent paper was read in full to assess its relevance to the CMOC being tested (See Table 3). If a parent paper was relevant to the current CMOC, it was advanced to the RRR screening phase. In some cases, a parent paper was also relevant to other CMOCs within the broader programme theory; in these instances, it was both progressed to RRR screening and marked as a *key informant paper* for future analysis. If a parent paper was not relevant to the current CMOC but held potential relevance for other CMOCs, it was designated solely as a *key informant paper*, to be revisited during the testing of those CMOCs. Finally, if a parent paper was found to be irrelevant to both the current CMOC and all other CMOCs, it was excluded from further analysis. Figure 4 provides a visual illustration of the decision tree used to guide the assessment of parent papers, outlining the pathways for inclusion, exclusion, or designation as key informant papers based on their relevance to the CMOC under review and/or the broader programme theory.

### Application example: parent paper relevance screen CMOC 1

**Fig. 5:**
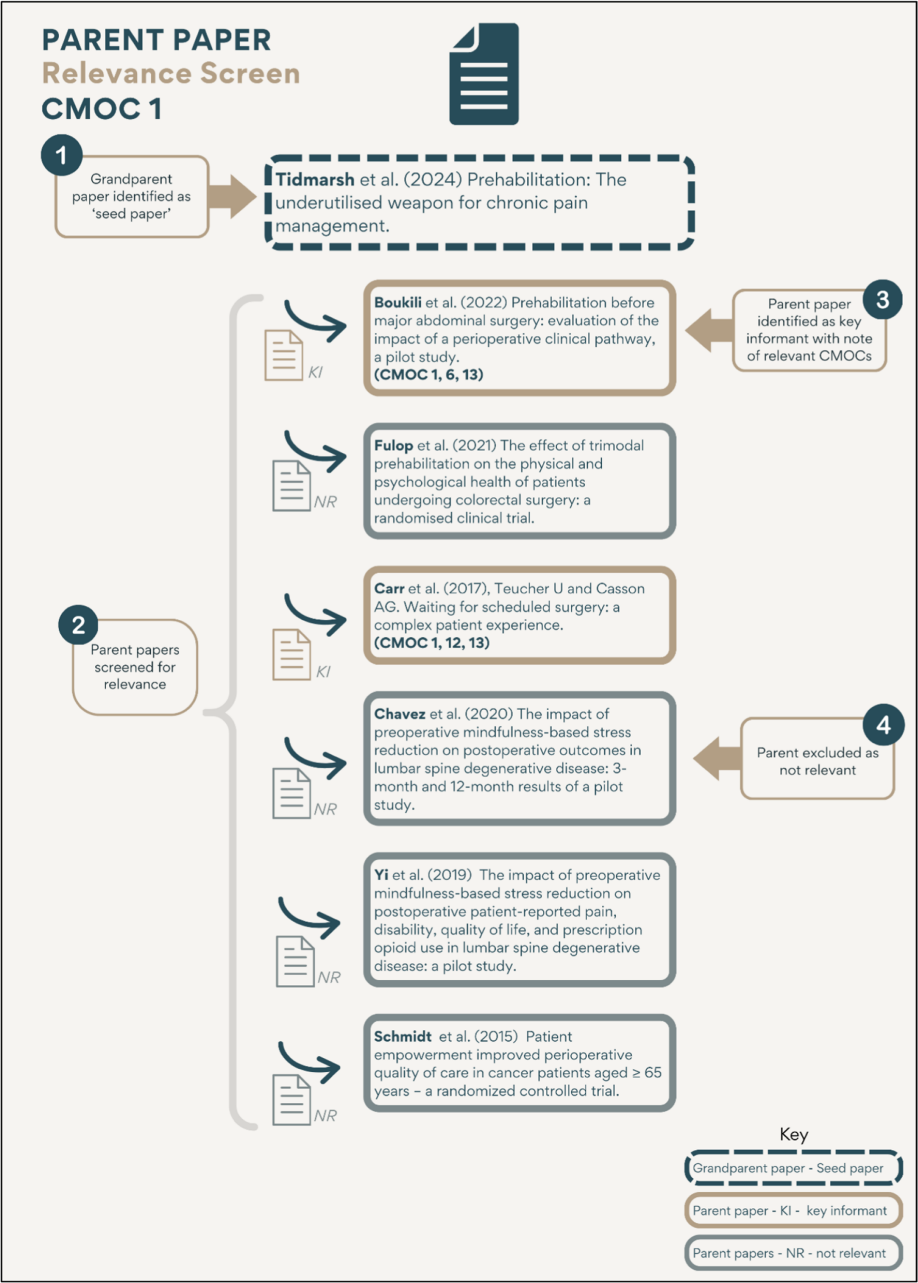
Parent relevance screen for CMOC 1

From the relevance screen of the nine grandparent studies identified for CMOC 1, one was categorised as a seed paper—a secondary study that cited potentially relevant primary studies. From this seed paper, six primary studies were identified and termed ‘parent papers’. These parent papers were read in full to assess their relevance using the same criteria applied during the grandparent screening stage. Of the six, four were excluded as not relevant to the current CMOC or any other CMOCs. The remaining two were found to be relevant both to CMOC 1 and to other CMOCs within the broader programme theory. These two parent papers were therefore included for RRR screening and simultaneously coded as key informant papers to be revisited in future CMOC analyses (see Fig. 5).

When a primary study is identified as relevant to more than one CMOC, it may seem redundant to code it as a key informant paper. It could be argued that, for expediency, the paper should go straight through to the RRR screening phase, tagged with hashtags for each relevant CMOC—bypassing the "key informant" categorisation. However, revisiting the paper when focusing on each new CMOC offers an opportunity to reassess its relevance in light of the current focus and the evolving data being analysed. Re-reading at this point allows researchers to refine their understanding and verify whether the study’s initial relevance remains valid or whether new insights have emerged that might affect its inclusion within another CMOC.

The act of revisiting a study is analogous to re-interviewing a participant in qualitative research after having spoken to additional participants. Insights gained from the other interviews may influence how you interpret the second conversation with the same person, allowing for a deeper understanding or a different perspective based on the new information [23]. Similarly, when revisiting a study in the context of a new CMOC, the interpretation of its relevance may evolve as it is considered alongside the findings from other studies. While this process may seem time-consuming, it tends to become increasingly efficient with each iteration. Since the study has already been reviewed, subsequent readings are generally lighter-touch providing an opportunity to add nuanced insights and annotations.

Revisiting a study when focusing on a new CMOC not only provides an opportunity to refine interpretations but also helps sensitise researchers to the emergence of demi-regularities—context-dependent patterns that reveal how mechanisms operate across different settings [24]. However, while searching for these demi-regularities, it is important to remain cautious of confirmation bias —the tendency to focus on evidence that supports an emerging idea while overlooking information that might contradict or complicate it [25]. This can lead to a reinforcing feedback loop where each item of supporting evidence strengthens belief in the prevalent idea and heightens sensitivity to similar patterns. This mirrors the ‘streetlamp effect’: searching only where the light is shining, rather than where the most meaningful evidence may lie [26]. Realist researchers are actively engaged in looking for rivalry and counterfactuals, which are fundamental for theory development [27]. By deliberately seeking alternative explanations and examining how different CMOCs might play out, researchers can refine their theories and ensure that they aren’t simply confirming pre-existing assumptions. While the challenge of confirmation bias cannot be fully eliminated, being aware of the broader aim of revisiting studies—testing, challenging, and refining emerging theories—helps mitigate this risk. This approach ensures that the review remains rigorous, balanced, and open to new insights, ultimately leading to a robust understanding of the relationships between context, mechanisms, and outcomes.

### Child papers

In some cases, primary studies referenced within included parent papers—referred to as child studies (second stage referrals)—may be potentially relevant to the CMOC under testing. The screening process for child papers follows the same procedure as parent papers. Decisions on whether to screen and include child studies can be made by the research team, typically guided by the extent of available data and the need for further evidence to test the CMOC in question. In our review, where the literature on nutrition in prehabilitation programmes for NC surgical candidates was relatively sparse, references from a few child studies were included. However, given time constraints and limited resources, a pragmatic decision was made not to extend the review to the child level for the remaining CMOCs, as the prevalent literature base was deemed sufficient. Authors thus face an ongoing decision on how many iterations (generations) are required, both in general, and for particular CMOCs. In doing so they need to decide whether to prioritise standardisation across CMOCs (following up the same number of levels for each CMOC) or to favour customisation to the specific needs of each CMOC (with different levels for each).

### Quality assessment

Quality assurance checks were applied at the parent study stage only, as this represented the point at which exclusion decisions posed the greatest risk to theory development. Formal quality assurance checks were not conducted at earlier stages of the screening process, including on records excluded by the AI-powered searches at the grandparent stage, as these stages functioned as relevance-based identification filters rather than points of quality appraisal. Potential gaps in retrieval were instead addressed through citation and reference checking of included papers.

**Fig. 6:**
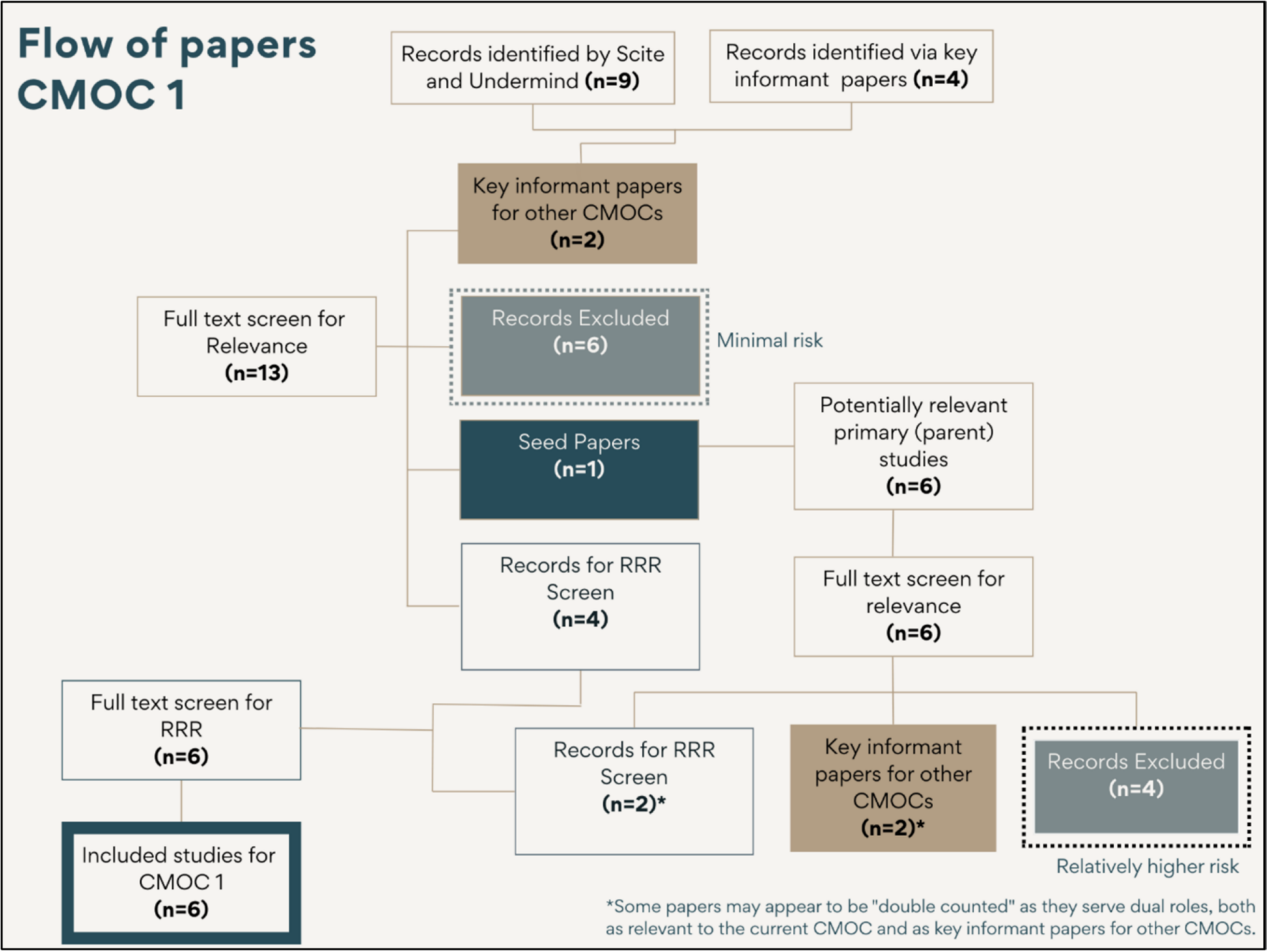
Study selection flow for CMOC *1* with exclusion points highlighted

At the parent study stage, we implemented a quality assurance check (see Fig. 6 and the box highlighted as ‘relatively high risk’). Since decisions about which papers to advance to RRR screening were based on judgments of relevance, ensuring a consistent and rigorous approach was important. Given that we were trialling this method, we adopted a cautious approach by having a second screener review 50% of the records excluded by the first reviewer for each CMOC. One disagreement was identified during this process, which was resolved through discussion and consensus. While this percentage may seem high, it proved feasible, as a total of 18 papers across all CMOCs were excluded at this stage, meaning the second screener only needed to assess nine papers. This additional check helped strengthen the reliability of our process. During this process, the second screener noted the importance of keeping a copy of all CMOCs readily available whilst screening to ensure that papers were assessed not only for relevance to the CMOC being tested but also for potential relevance to other CMOCs within the broader framework.

Figure 6 uses CMOC 1 as an example to illustrate the flow of studies included in the review, starting with the grandparent studies retrieved through the AI-powered searches using Scite, Undermind, and key informant papers. The diagram traces the path to the parent studies, highlighting key points at which studies were excluded from the review. Two boxes with dashed outlines indicate these exclusion points. The second exclusion box labelled ‘relatively high risk,’ marks the stage where a second reviewer was introduced for quality appraisal, as described earlier in the methodology. Such an approach harmonises with recent approaches to quality assessment, particularly for rapid review methods, where previous arbitrary models of double checking are increasingly being replaced with strategies specifically targeting stages considered of higher risk [28]. Similar flow diagrams were created for all 14 CMOCs included in the programme theory, ensuring consistency and transparency throughout the study selection process.

### Relevance, richness and rigour appraisal

The RRR screening process served as a transparency step rather than a strict eligibility filter. By this stage, all papers had already undergone an initial relevance screen, ensuring they were relevant to one or more CMOCs. As a result, there were no exclusions, and the RRR screen focused on assessing the confidence with which conclusions could be drawn from each paper. Each paper was evaluated against three independent criteria: relevance, richness, and rigour, with scores of high, moderate, or low assigned to each (see supplementary file 3). While papers could theoretically be excluded if they scored low across all three criteria, in practice, the RRR screen primarily aimed to clarify the strength of the evidence and highlight any limitations. For example, papers with high relevance but lower richness or rigour were still included, but their limitations were explicitly acknowledged to ensure transparent and nuanced interpretation of the findings. This approach aligns with the guidance provided by Dada et al. [21], which emphasises transparency in the evaluation of evidence to ensure clarity regarding the confidence in findings and conclusions drawn from studies.

## Discussion

This case study demonstrates how AI-powered tools can enhance literature searching by retrieving conceptually rich and contextually relevant studies that might be overlooked using conventional title- and abstract-based searches. Using tools such as Scite and Undermind within a structured workflow— including seed papers, key informant papers, and a grandparent–parent–child classification system— allowed us to improve the conceptual quality of the retrieved studies in terms of rigour, relevance, and richness. Although developed for a realist review, this approach can be applied more broadly to literature reviews where hard-to-find or conceptually rich evidence is important. By extending searches beyond conventional strategies, AI-assisted methods can complement human judgement and support the identification of studies that inform theory development and refinement.

Conventional keyword searches often rely on the researcher's vocabulary aligning with that used in titles, abstracts, or indexing terms. This limitation can hinder the discovery of relevant literature that uses alternative language or conceptual framing. In contrast, AI-powered tools like Undermind and Scite use natural language processing (NLP) to assess full-text similarities and conceptual relationships. This allowed us to retrieve literature aligned with the theoretical underpinnings of our CMOCs—even when key terms were absent—thereby improving conceptual retrieval beyond the capabilities of traditional search strategies. Importantly, these tools were deployed within tightly defined conceptual boundaries (i.e., specific CMOCs), rather than broader population–intervention–comparison–outcome (PICO) queries, making them particularly suited to realist inquiry. For example, when testing CMOC 1—which proposed that being listed for surgery provides relief and empowerment for patients who have been advocating for their health, leading to increased motivation to engage in prehabilitation—the AI tools retrieved studies examining patient experiences of surgical waiting lists, the psychological impact of having to ‘advocate’ for themselves, and motivation for pre-operative preparation. These papers were conceptually aligned with our CMOC but did not necessarily use terms like 'empowerment' or 'relief' in their titles or abstracts.

AI-powered tools enhanced our ability to identify relevant papers for testing our CMOCs. Conventional keyword searches rely on specific terms and phrases input by the researcher matching either those used by the author in their title or abstract or those used by an indexer to enhance retrieval. Not only does this limit the search to the researcher's knowledge and their associated vocabulary but it also misses greater retrieval opportunities offered by the richness of full text retrieval. As a result, these searches may miss conceptually related literature that uses different terminology or phrasing. In contrast, tools such as Undermind and Scite employ natural language processing techniques to identify papers that align conceptually with the search query, even when the exact terms or synonyms are not present. By drawing on a broader knowledge base, these tools offer a nuanced approach that extends beyond keyword searches. This capability, broadening retrieval but only within very tight search parameters (the language associated with a CMOC, not a broader PICO review question) was particularly valuable for our realist review, as we sought literature to test concepts, identify mechanisms, and understand important contextual factors.

While AI tools like Scite and Undermind improved the conceptual precision of our screening process, they did not replace the interpretive work required in a realist review. Their ability to identify semantically relevant papers and provide summarised insights helped surface data that may have been overlooked using traditional keyword searches. However, the task of making sense of these findings —through retroductive and abductive reasoning [29] remained firmly with the research team. This interpretive work was essential in testing, refining, and refuting our CMOCs. For instance, when papers discussed patients wanting to 'take an active role' during the waiting period, we had to interpret whether this genuinely evidenced our theorised mechanism of empowerment, or whether it reflected a different psychological process entirely. Our findings therefore support the view increasingly reflected in the literature: AI is not a substitute for human judgement but a valuable adjunct to it. Studies such as Ge et al. [30] and Gwon et al. [31] highlight similar dynamics in other evidence synthesis contexts, where AI accelerates routine tasks like screening but requires human oversight for rigour and contextual interpretation. Feng et al. [32] further emphasise this by demonstrating that AI tools often achieve high recall (i.e., successfully identifying most relevant papers), but can vary in precision—reinforcing the need for critical human appraisal. As such, the integration of AI tools can enhance the scope and sensitivity of realist reviews, but only when grounded in thoughtful, theory-driven human interpretation.

### Challenges and limitations

While this manuscript is based on a single case study, using a detailed, single-case application to introduce and demonstrate a new methodological approach is a common practice. Much of the methodological evidence for information retrieval in systematic reviews is built on individual case studies rather than large comparative studies [33–35] and this is recognised as a standard approach for advancing search methodology. Nonetheless, there are several challenges and limitations in this case study that are worth noting, and the approach requires further testing and evaluation in other contexts and with other research teams to assess its broader applicability and robustness.

AI-powered tools operate within the constraints of their underlying algorithms, and the lack of transparency in the coding of these algorithms may introduce biases. For instance, the relevance scoring could reflect limitations or preferences embedded in the training data or algorithmic design potentially leading to certain types of evidence being emphasised over others. Additionally, these tools may have limited access to certain databases or publication types, such as grey literature or very recent publications, which could result in gaps in the literature covered. Human reviewers are encouraged to be reflexive about their influence on research processes, AI systems are not – although some researchers have begun experimenting by scripting AI systems to identify their own limitations!

Furthermore, as these tools are relatively new, limited evidence exists for their performance against librarian-led search strategies, especially in the context of realist reviews. Realist reviews prioritise identifying conceptually relevant literature over exhaustive searches [5], so the effectiveness of AI-powered tools should be evaluated based on their ability to capture studies that meaningfully contribute to programme theory development, using criteria such as relevance, richness, and rigour [21]. While this study did not conduct a head-to-head comparison with traditional or baseline literature review methods using the same keywords, the value of the AI-assisted approach lies in retrieving conceptually rich and contextually relevant studies, prioritising quality over quantity. Empirical evaluation against conventional searches remains a potential avenue for future research**.** Despite the promise of AI-powered tools, they still require a degree of methodological expertise to use effectively. Researchers with a solid understanding of realist methodology—or working within a team with such experience—may be better equipped to formulate appropriate search queries and critically assess the conceptual relevance of retrieved studies. AI-powered tools are a useful adjunct to traditional search methods, helping to streamline the process. However, we would argue that they cannot replace the nuanced judgement and interpretation essential to realist review. Specifically, the time overhead required to oversee and validate their otherwise credible-looking output should not be overlooked.

While AI-powered tools can save time in searching and assembling the literature, time spent categorising papers may offset these gains. The multiple screening stages—such as categorising grandparent, parent, and child papers—demand significant researcher time and effort. However, this challenge arose primarily because this approach was novel and developed iteratively; with clearer instructions in place, it is likely to be quicker and more efficient in future applications. Our classification system and decision trees will require ongoing refinement and validation to ensure their continued credibility across different contexts.

A limitation regarding accessibility stems from our decision to use AI tools which require paid subscriptions for full functionality. However, the use of specialised, commercial software is a standard practice in modern academic research (e.g., licensed statistical or reference management packages). Furthermore, many features of Scite and Undermind could be used in the free or 'lite' versions of these applications, thereby mitigating the access barrier for resource-constrained researchers.

Finally, a key limitation of this review was the absence of quality assurance checks during the initial and grandparent screening stages. However, at the parent screening stage—where the risk of excluding rich and relevant studies was considered greater—we introduced a quality check, with a second reviewer screening 50% of excluded studies. While arguably risk averse, this step reflects a commitment to maintaining rigour in the screening process. Other research teams may wish to consider introducing similar checks earlier, depending on the stage at which key decisions are made and the level of confidence in the tools used.

## Conclusion

This case study offers a methodological resource for researchers seeking to integrate and make the most of AI-powered literature search tools in support of realist reviews. Searching for nuanced concepts to build explanatory theories is fundamental to realist synthesis, but it often involves navigating large volumes of literature in pursuit of elusive 'mechanisms' or key contextual factors.

By demonstrating the application of tools such as Scite and Undermind, this case study shows how AI can support targeted, conceptually driven searches—enabling researchers to access rich and relevant data. However, as with any tool, their value lies in how they are used. This is especially true for AI-powered searches, which are still relatively new. We are continuing to learn how best to use these tools effectively, and their role is to complement—not replace—the judgment and interpretive thinking of the researcher.

The methods outlined in this case study offer a practical framework for selecting, organising, and appraising search results in a transparent and robust way, contributing to the ongoing development of realist review methodology. While not intended as a definitive blueprint, this case study offers a foundation for others to build upon, test, and refine as methodological practices continue to evolve.

## Supplementary Information

Supplementary Material 1.

Supplementary Material 2.

Supplementary Material 3.

## Data Availability

The authors confirm that key materials supporting the findings of this study are provided within the article and its supplementary materials. Additional flow diagrams and related data are available from the authors upon reasonable request.

## Abbreviations

AI: Artificial Intelligence
CMOC: Context-Mechanism-Outcome configuration
RRR: Relevance, Richness, Rigour
